# Effect of thematic map misclassification on landscape multi-metric assessment

**DOI:** 10.1007/s10661-015-4546-y

**Published:** 2015-05-05

**Authors:** William J. Kleindl, Scott L. Powell, F. Richard Hauer

**Affiliations:** Flathead Lake Biological Station and Montana Institute on Ecosystems, University of Montana, Missoula, MT 59812 USA; Department of Land Resources and Environmental Sciences, Montana State University, Bozeman, MT 59717 USA

**Keywords:** Landscape pattern, Land-use intensity, Thematic classification accuracy, Landscape metrics, Bias, Remote sensing

## Abstract

Advancements in remote sensing and computational tools have increased our awareness of large-scale environmental problems, thereby creating a need for monitoring, assessment, and management at these scales. Over the last decade, several watershed and regional multi-metric indices have been developed to assist decision-makers with planning actions of these scales. However, these tools use remote-sensing products that are subject to land-cover misclassification, and these errors are rarely incorporated in the assessment results. Here, we examined the sensitivity of a landscape-scale multi-metric index (MMI) to error from thematic land-cover misclassification and the implications of this uncertainty for resource management decisions. Through a case study, we used a simplified floodplain MMI assessment tool, whose metrics were derived from Landsat thematic maps, to initially provide results that were naive to thematic misclassification error. Using a Monte Carlo simulation model, we then incorporated map misclassification error into our MMI, resulting in four important conclusions: (1) each metric had a different sensitivity to error; (2) within each metric, the bias between the error-naive metric scores and simulated scores that incorporate potential error varied in magnitude and direction depending on the underlying land cover at each assessment site; (3) collectively, when the metrics were combined into a multi-metric index, the effects were attenuated; and (4) the index bias indicated that our naive assessment model may overestimate floodplain condition of sites with limited human impacts and, to a lesser extent, either over- or underestimated floodplain condition of sites with mixed land use.

## Introduction

Advances in ecological assessment tools designed to assist in the management of aquatic systems at broad spatial scales have paralleled increased access to remote-sensing products and advances in geographic information processing. Remote-sensing products, such as thematic maps from Landsat or orthorectified imagery, provide the necessary baseline data to link alterations in landscape structure to perturbations in ecosystem functions at these large scales. These remote-sensing data have known errors that should be, and generally are, clearly articulated in the metadata or associated accuracy reports. However, efforts to incorporate these errors into ancillary products, such as assessment tools, remain limited (Shao and Wu 2008). Ignoring the implications of these known errors on the results of assessment models potentially affects the level of confidence that resource managers have in the information the tools provide, and ultimately determines the extent to which the tool is used.

Indicator-based ecological assessment models have been developed to provide decision and policy makers with the needed ecological information for determining resource management decisions, communicating those decisions to the public, and developing rules to protect resources (Turnhout et al. 2007; Dramstad 2009). In reviews of contemporary aquatic assessment models, the multi-metric index (MMI) was the predominant indicator-based approach (Diaz et al. 2004; Fennessy et al. 2004; Böhringer and Jochem 2007). MMI tools developed for assessments at watershed scales (Brooks et al. 2004; Tiner 2004; Weller et al. 2007; Meixler and Bain 2010), regional scales (e.g., Reiss and Brown 2007; Collins et al. 2008), or compiled to provide national scale assessments (USEPA 2013) commonly use remotely sensed data and imagery to develop scale-appropriate metrics (Fennessy et al. 2007). While cartographic data generally follow standardized reporting guidelines that articulate known uncertainties inherent in the product (Foody 2002), incorporating these known uncertainties into MMI tools is rare (Fore et al. 1994; Whigham et al. 1999; Stein et al. 2009) and tends to be absent in the assessment implementation and reporting phase (e.g., Smith et al. 1995; Hauer et al. 2002; Klimas et al. 2004; Collins et al. 2008).

Ideally, a well-constructed ecological MMI model is designed to facilitate resource decisions by providing straightforward analyses of ecological data to enable translation to management applications (Barbour et al. 1999). However, addressing the implications of uncertainty in these tools can be complex. The challenge is to provide a pathway to incorporate known uncertainties from multiple scale-appropriate data sources into an assessment tool used by planners, policy makers, lawyers, and scientists. In this paper, we address two questions to meet this challenge: How sensitive is a landscape-scale multi-metric index to error from input data (specifically thematic land-cover misclassification)? What are the implications of this uncertainty for resource management decisions?

## Methods

To answer these questions, we developed a multi-metric index that uses thematic Landsat data to provide an assessment of floodplain conditions along 250 km of the Flathead River in northwestern Montana, USA. Typical of most multi-metric indices, our initial assessment did not account for misclassification errors within the thematic map and produced metric and index scores that were considered naive. We then provided an error simulation model to incorporate known map classification error into our multi-metric assessment tool by developing multiple potential map realizations based on classification probabilities and potential spatial correlations. We applied our MMI to each realization to bind the potential stochasticity of the classification error (noise) into a distribution of potential assessment scores. We then compared this distribution to the naive score to determine potential bias and the implications of that bias on management decisions.

### Study area and site selection

**Fig. 1:**
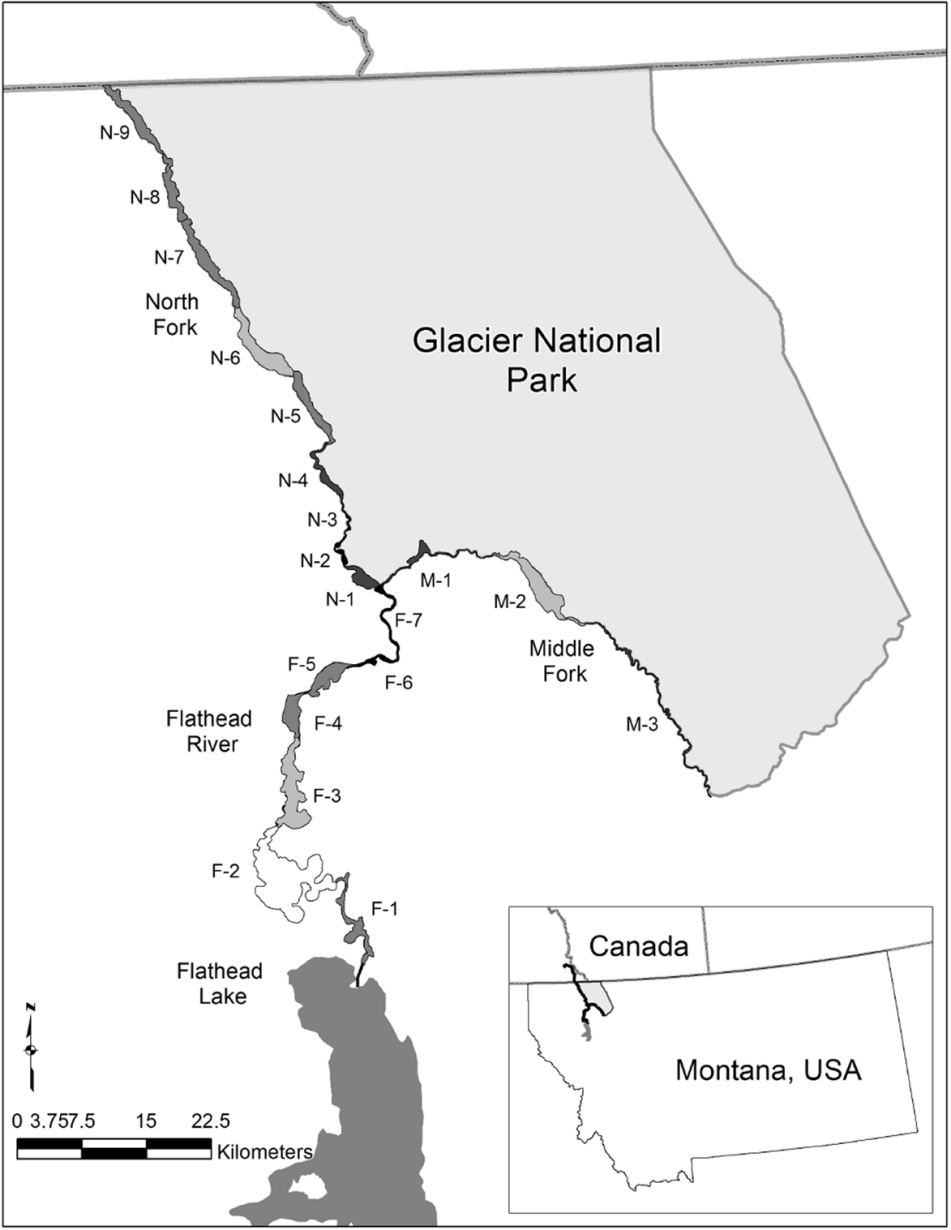
Location of study area and the 19 floodplain assessment sites. N-1 through N-9 are on the North Fork of the Flathead, M-1 through M-3 are on the Middle Fork, and F-1 through F-7 are sites on the Flathead River main stem

**Fig. 2:**
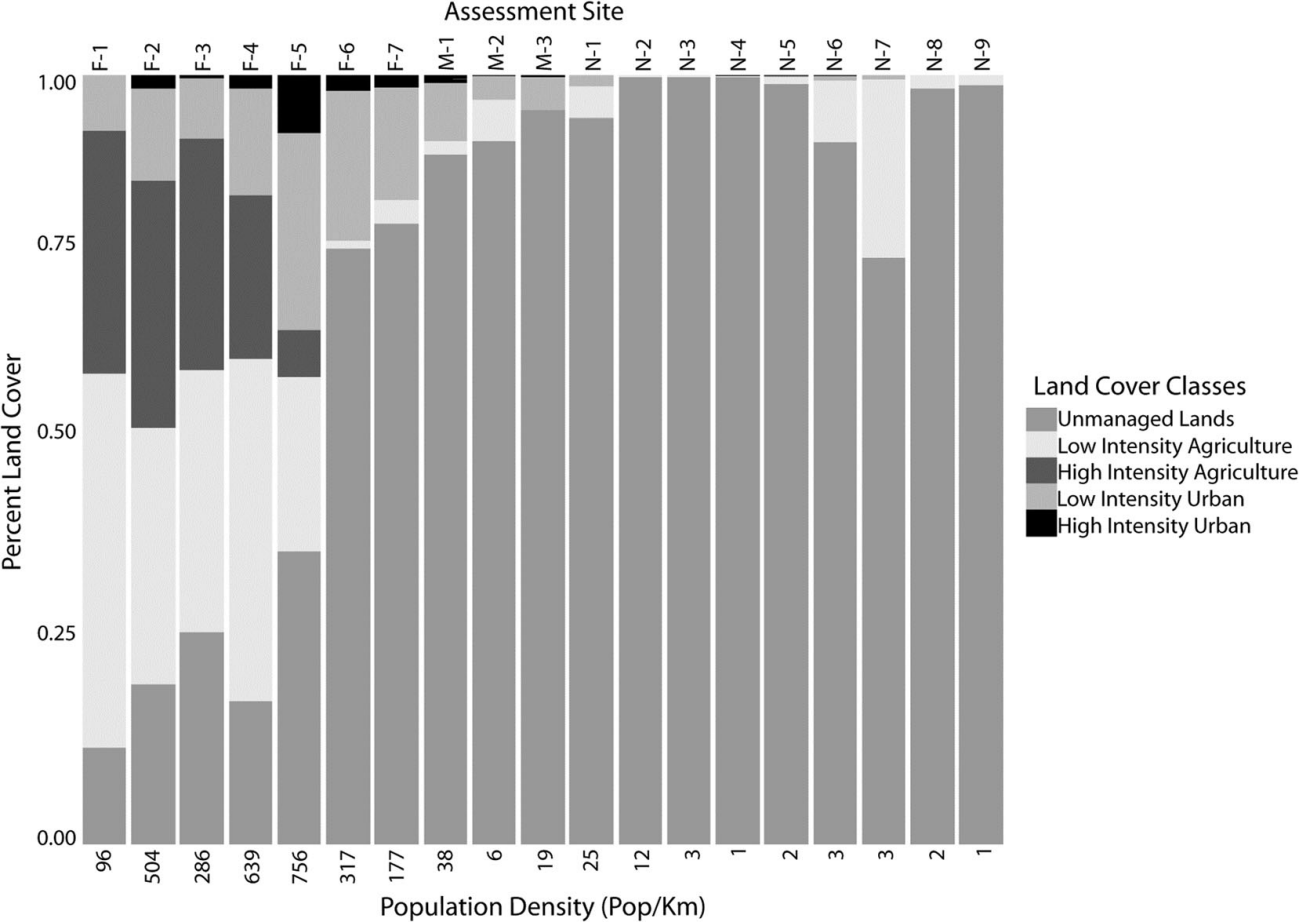
Percent cover of land-cover classes and population density (Montana State Library 2011) for each assessment site (floodplain and buffer area combined)

Our assessment model was centered on the Flathead River system above Flathead Lake within northwestern Montana, USA and included portions of the North Fork, Middle Fork, and main stem of the Flathead River (Fig. 1). The study area consisted of land use and land cover (LULC) typical in floodplains of larger rivers in the Northern and Canadian Rocky Mountains (Fig. 2). The North Fork of the Flathead River has its headwaters in southeastern British Columbia, Canada and enters the study area as it crosses the U.S. border. Within the study area, the river flows 93 km south-by-southeast along the northwest boundary of Glacier National Park (GNP) through a broad U-shaped valley with expansive low-gradient montane alluvial floodplains that are predominantly covered with forest and grasslands (simply called “unmanaged lands” here) and occasional pasture, as well as urban and exurban development (called “managed lands” here). The Middle Fork has its headwaters in the Bob Marshall Wilderness Area and enters the study area as it emerges from the wilderness complex and meets the southwest GNP boundary. Within the study area, the Middle Fork flows 70 km through a series of confined and unconfined reaches within a narrow valley that also contains U.S. Highway 2, the Burlington Northern Santa Fe Railroad transportation corridor, and the small town of West Glacier, MT at the southwestern tip of GNP. The main Flathead River channel begins at the North and Middle Fork confluence and flows about 86 km southerly leaving the study area as it enters the 480 km^2^ Flathead Lake. Along the way, this sixth-order river leaves the confined forested slopes and enters a broad piedmont valley floodplain consisting of agricultural, urban, and exurban development interspersed with floodplain forest.

Nineteen assessment areas were selected based on continuous floodplain reaches separated by geomorphic constrictions on the river valley (Fig. 1): nine sites on the North Fork (numbered N-1 through N-9 from downstream to upstream), three sites on the Middle Fork (M-1 through M-3), and seven on the Flathead River main stem (F-1 through F-7). These sites consisted of both broad alluvial depositional areas typically associated with floodplain ecosystems and confined reaches with limited floodplain. Local biological diversity of river and floodplain systems is strongly influenced by surrounding land-use at several scales including local buffers (Morley and Karr 2002; Allan 2004; Pennington et al. 2010). To account for local land-use impacts adjacent to floodplain habitats, we established a 1-km buffer to the entire floodplain area and delineated 19 buffer assessment sites perpendicular to the outer edge of each floodplain assessment site. The assessment sites and their buffers collectively have land use that ranges from forest to agriculture to urban, with human population densities ranging from 1 to 636 people per kilometer (Montana State Library 2011, Fig. 2).

The 19 assessment sites were digitized in ArcGIS 10.0 (ESRI 2011) with the assistance of 2005 background orthoimagery from the USDA National Agriculture Imagery Program (NAIP; USGS 2014). Digitalization was aided with a 30-m digital elevation map (USGS 2013), visual assistance from oblique views within Google Earth’s 3-D models (Google Earth 2013), oblique imagery from aerial reconnaissance, and multiple site visits. Unless otherwise stated, all data collection, organization, and subsequent analyses were conducted in ArcGIS 10.0 (ESRI 2011) and the R system for statistical computing (R Core Team 2013).

### Multi-metric index case study

Multi-metric indices are composed of qualitative measures of the condition of biotic and abiotic structural attributes that, in combination, support ecological function or maintain ecosystem integrity. To create metrics, a score is assigned (e.g., 0-1, 1-100) to the attribute; to create an index, these metrics are combined in a manner that best describes the attribute’s relative contribution to system function or integrity. The metric scores and index model are based on reference data, literature, and expert opinion of model developers to assess specific ecosystem aspects important to management (e.g., habitat, ecosystem services, or overall function). In practice, a robust landscape-scale floodplain assessment model may incorporate attributes from multiple spatial datasets, such as road densities, wetland inventories, soil databases, elevation, slope, and human population density. In this case study, for illustrated purposes only, we developed a simplified MMI with metrics derived from a single thematic map, the 2006 National Land Cover Database (NLCD), to specifically address aspects of uncertainty that arise from a single data source. For this case study, we followed MMI general protocols (e.g., Smith et al. 1995; Barbour et al. 1999; Stoddard et al. 2008) to develop a simplified tool to address the overall condition of native floodplain cover.

NLCD thematic classified maps were developed for the conterminous United States by a coalition of U.S. agencies (MRLC 2013) using Landsat Thematic Mapper (TM) data for the 1992 map (Vogelmann et al. 2001) and Landsat Enhanced Thematic Mapper+ (ETM+) data for maps from years 2001 (Homer et al. 2007), 2006 (Fry et al. 2011), and 2011 (Jin et al. 2013). From 2001 on, NLCD used a decision-tree-based supervised classification approach to create a land-cover classification scheme at a spatial resolution of 30 m, followed by the aggregation of pixels to achieve a minimum mapping unit of approximately 0.40 ha, to assign pixels to one of 16 classes (Homer et al. 2004, 2007). The supporting NLCD literature also provided accuracy assessments in the form of a confusion matrix containing overall, producer’s, and user’s accuracy calculations that clearly articulated map classification error (MRLC 2013). These products do not require the map user to collect or process additional data; therefore, we applied the same limitation and did not collect additional site-specific accuracy data for this study beyond what was supplied with the NLCD product. Here, we used the 2006 NLCD classified map from Path 41 and Row 26 (MRLC 2013) clipped to our floodplain and buffer polygons for each of the 19 assessment areas.

#### Landscape metrics

For our landscape-scale MMI, we derived two metrics from the 2006 NLCD data: (1) a perturbation metric that assessed land-use intensity and (2) a fragmentation metric that measured land-cover configuration. Each metric was first calculated for the buffer and floodplain areas and then subsequently combined into the assessment index.

##### Perturbation metric for buffer and floodplain areas (*Met*_*BP*_ and *Met*_*FP*_)

The aerial extent of human-altered land cover within an assessment site is a commonly used indicator of the site’s overall anthropogenic stressors (O’Neill et al. 1999; Tiner 2004; Brown and Vivas 2005). To extract this information from the NLCD categorical maps, 16 land-cover classes from the original map were binned into five major land-use groups that best represented the anthropogenic land-use disturbance gradient found within the study area (Table 1): (1) unmanaged lands, (2) low-intensity agriculture, (3) high-intensity agriculture, (4) low-intensity urban, and (5) high-intensity urban. Because we were developing an assessment of native floodplain cover condition, we treated all such cover as a single “unmanaged land” cover class.

**Table 1:** NCLD cover types binned to reflect a gradient of major land-use categories and the weighted sub-score assigned to each category, reflecting the gradient of land-use intensity used in the perturbation metric

Buffer and floodplain land-use criteria	Weighted sub-score
Unmanaged land cover: land-cover characteristic of Rocky Mountain floodplain systems, which include open water, forest, shrub, herbaceous, and wetlands cover classes. NCLD Codes 11, 12, 41, 42, 43, 52, 71, 90, and 95	1.0
Low-intensity agriculture: herbaceous areas used for pasture and hay. NCLD code 81	0.8
High-intensity agriculture: cultivated row crops. NCLD code 82	0.5
Low-intensity urban: developed open space and low-intensity developed lands. NCLD codes 21 and 22	0.2
High-intensity urban: barren ground (predominantly gravel mines, but also includes to a much lesser extent cobble), as well as medium- and high-intensity developed lands. NCLD codes 23, 24, and 31	0.0

For the purposes of this study, each of the five land-cover groupings was subjectively weighted, based on expert opinion, to represent the degree of divergence from land cover that was characteristic of unperturbed conditions typical of Rocky Mountain valleys (Table 1). Within each assessment area, buffer (*Met*_*BP*_) and floodplain (*Met*_*FP*_) areas were separately scored using Eq. 1:

1 $$ Met=\frac{{\displaystyle {\sum}_{x=1}^x\left(\sum {C}_{Lx}\ast {w}_{Lx}\right)}}{N} $$

where the metric score (*Met*) for the buffer or floodplain assessment area is equal to the total raster cells per cover class (*C*_*Lx*_) multiplied by the weighted sub-score for that class (*w*_*Lx*_) from Table 1, summed across all classes (*x*), then divided by the total cell count (*N*) of the assessment area to obtain a score that ranges between 0.0 and 1.0. The closer the metric score is to 1.0, the more likely the area has land-cover characteristics of an undisturbed system. A score closer to 0.5 represents agricultural land cover, and 0.0 represents an area dominated by urban land-cover.

##### Habitat fragmentation metric for buffer and floodplain areas (*Met*_*BF*_ and *Met*_*FF*_)

The above perturbation metrics assess the extent of human alteration. However, two sites with the same relative abundance of unmanaged land could provide different levels of structural support for native biota depending on the degree of fragmentation (Vogt et al. 2007). Our fragmentation metric measured the degree of continuity within landscape patterns (Gustafson 1998; O’Neill et al. 1999). We used a morphological spatial pattern analysis (MSPA) GIS tool (Joint Research Station 2014) to identify the extent of contiguous and isolated patches, perforations within those patches due to agriculture and urban areas, and the amount of edge between these managed and unmanaged lands. The MSPA input required a binary map consisting of unmanaged lands from Table 1 and a cover type called managed lands that was created by binning all agriculture and urban land-cover types in Table 1. The output of the MSPA tool was a map containing a mutually exclusive set of seven patch and edge structural classes within the floodplain and its buffer (Vogt et al. 2007; Soille and Vogt 2009; Suarez-Rubio et al. 2012): (1) core areas, (2) patch edges, (3) loops, (4) bridges, (5) branches, (6) islets, and (7) managed lands. Each structural class was subjectively assigned a weighted sub-score based on an expert opinion that represented the degree of fragmentation or edge (Table 2). The structural class assignments were then clipped to each buffer and floodplain assessment site.

**Table 2:** Description of structure categories of the fragmented landscape and the weighted sub-score assigned to each category, reflecting the gradient of habitat quality used in the fragmentation metric

Fragmentation structure	Weighted sub-score
Core areas—pixels of unmanaged lands inside of a defined 90-m (3 pixels) wide patch width (pixel value from a post MSPA map are 17, 117)	1.0
Patch edge—pixels of unmanaged lands that are comprised of patch edge adjacent to managed land-cover type (MSPA pixel value 3, 5, 35, 67, 103, 105, 135, 167)	0.8
Loop—pixels that connect one patch of core unmanaged lands to the same core area and are completely made up of edge (MSPA pixel value 65, 69, 165, 169)	0.6
Bridge—pixels that connect one patch of core unmanaged lands to another core area and are completely made up of edge (MSPA pixel value 33, 37, 133, 137)	0.6
Branch—pixels that emanate from core, bridge, or loops into managed lands and are completely made up of edge (MSPA pixel value 1, 101)	0.4
Islet—pixels of unmanaged lands within a patch of managed lands that is completely made up of edge (MSPA pixel value 9, 109)	0.2
Managed lands—all remaining pixels (MSPA pixel value 0, 100)	0.0

The fragmentation metric score for both the buffer (*Met*_*BF*_) and floodplain (*Met*_*FF*_) was calculated using Eq. 1, where total raster cells per MSPA structural class (*C*_*Lx*_) at each site were determined and multiplied by the weighted sub-score (*w*_*Lx*_) from Table 2. The closer the metric score was to 1.0, the more the likely the area had contiguous land-cover characteristic of an undisturbed system; the closer to 0.0, the more likely the area had a contiguous cover of managed land.

#### Flathead river floodplain condition index

Finally, we applied the index model (Eq. 2) to calculate the Flathead River floodplain habitat condition based on land-use intensity and habitat fragmentation:

2 $$ \mathrm{Index} = \left(\left(\left( Me{t}_{BP} + Me{t}_{BF}\right)/2\right) + Me{t}_{FP} + Me{t}_{FF}\right)/3 $$

The condition of the buffer influences the condition of the floodplain (Allan 2004); therefore, we first averaged the buffer metrics (*Met*_*BP*_ and *Met*_*BF*_). We then added that product to the floodplain metrics (*Met*_*FP*_ and *Met*_*FF*_) and averaged the final product to provide a score between 0 and 1. Scores closer to 0.0 represented a disturbed landscape and scores closer to 1.0 represented an intact ecosystem in excellent condition. This MMI provided a naive estimate of ecological conditions and was, in essence, the data collection component of the methods. The following data analysis methods address the impact of input map error on these results.

### Data analysis

We addressed map misclassification effects on the MMI results by first reducing the map error from the original NLCD 2006 map (MRLC 2013) where possible without additional data collection. Then we incorporated the remaining unavoidable error into the metrics and index. Finally, we tested the bias of the naive MMI results when we incorporated this remaining error.

#### Reducing uncertainty

Two maps were created for the study area: (1) a land-use map used to assess the two perturbation metrics and (2) a binary map used to assess the two fragmentation metrics. Each map was created by aggregating thematic classes from the original data, thereby decreasing the thematic resolution of the original land cover classification. We aggregated the confusion matrix from the original accuracy assessment to create new confusion matrices for each new map. We also calculated the overall accuracy indices (Congalton and Green 2008) and compared these to the original 2006 NLCD accuracy indices (Fry et al. 2011) to determine the effects of changing thematic resolution on error.

#### Error simulation model

Simulation models that use available confusion matrix information to account for misclassification error were developed in the 1990s (Fisher 1994; Hess and Bay 1997; Wickham et al. 1997). These models convert confusion of matrix user’s or producer’s accuracy information to a matrix of probabilities that inform the likelihood that an individual pixel is misclassified (Hess and Bay 1997). To meet the needs of potential resource managers, we created a matrix of probabilities based on user’s accuracy. This “User’s Probability Matrix” (UPM) is the proportion of locations classified in the map as *k*_*i*_ (mapped pixels in class (*k*) found across all reference columns *i* through *n*) in a confusion matrix. For example, a hypothetical accuracy assessment was conducted on 100 randomly selected pixels mapped as forest (*k*). These mapped pixels were checked against ground reference data; 90 pixels were determined to be forest (*k*_1_) and the remaining 10 were grassland (*k*_2_). From these hypothetical accuracy data, our UPM would assume that there was a 90 % probability that any forested pixel in our map was actually forest and a 10 % probability that it was actually grassland. Following this, we created UPMs for all thematic classes from the confusion matrices of both the perturbation land-cover and binary fragmentation input maps (Tables 3 and 4).

**Table 3:** User probability matrix represents the likelihood that a pixel on the perturbation map is actually one of several ground-reference pixels (UPM is used to support the perturbation metric simulation)

	Reference (*k*_1_–*k*_5_)				
Map (*k*)	Unmanaged lands	Low-intensity agriculture	High-intensity agriculture	Low-intensity urban	High-intensity urban
Unmanaged lands	93.10	3.24	1.68	1.78	0.20
Low-intensity agriculture	16.32	77.29	1.25	4.88	0.26
High-intensity agriculture	4.02	5.50	88.05	2.40	0.03
Low-intensity urban	19.96	5.10	5.14	65.40	4.40
High-intensity urban	18.32	0.81	0.27	8.31	72.29

**Table 4:** User probability matrix represents the likelihood that a pixel on the fragmentation map is actually one of several ground-reference pixels (UPM is used to support the fragmentation metric simulations)

	Reference (*k*_1_–*k*_2_)	
Map (*k*)	Unmanaged lands	Managed lands
Unmanaged lands	93.10	6.90
Managed lands	10.39	89.61

In geographic studies, it is accepted that “nearby things are more similar than distant things” (Tobler 1970) and is the basis of most spatial autocorrelation studies and tools (Goodchild 2004). Because we did not collect additional data, we could not assess the spatial structure of the error. Therefore, in the second step of our simulation model, we incorporated an autocorrelation filter proposed by Wickham et al. (1997), which assumes an overall 10 % difference in the classification error between the edge and interior pixels of a land-cover patch as a result of the influence of correlation between classified pixels (Congalton 1988). Applying a 10 % spatial autocorrelation filter decreased the likelihood of classification errors within patches (salt and pepper errors) and also increased the likelihood of misclassifications near patch boundaries that were generally associated with errors resulting from mixed pixels and spatial misregistration. We applied a 3 × 3 moving window to locate the patch interior and edge in the two metric input maps. We then created filters that decreased the effects of the UPM by 5 % for the interior pixels and increased the UPM by 5 % at the patch edge. Additionally, we tested the Wickham et al. (1997) 10 % autocorrelation modification against a 20 % gradient to determine sensitivity of the simulated index results to these modifications.

Finally, to account for the remaining classification error, we applied a confusion frequency simulation Monte Carlo model (CFS) that takes advantage of the a priori error probabilities in the UPMs to create stochastic realizations of our perturbation and fragmentation input maps (Fisher 1994; Wickham et al. 1997). For each simulation, the CFS (1) identified cover class *k* assigned to an individual map pixel, (2) drew a random variable from a uniform (0, 1) distribution, (3) adjusted the random variable with the autocorrelation filter, (4) determined the probabilities with all reference classes (*k*_1_–*k*_*n*_) associated with cover class *k* in the UPM, (5) assigned reference class *k*_*i*_ to the output simulation for that cell based on the modified random value and user probability, and (6) repeated this process for all remaining classes to create a single simulated realization of the map. The CFS was conducted under the assumptions that each pixel was eligible for selection, and each pixel was independently classified (Hess and Bay 1997). With this process, 1000 Monte Carlo simulations were created for each map. For the fragmentation map, the MSPA tool was applied to each simulated output.

#### Metric and index error assessment

Following each simulation, we calculated a buffer and floodplain score for each metric (Eq. 1) and total index score (Eq. 2), generating a distribution of 1000 potential metrics and condition scores. It was assumed that each Monte Carlo simulation was an independent sample of that classification error and that the distribution of simulated metric and index scores represented a raw stochastic sample of the error model behavior. We did not make assumptions about the structure of the simulated distributions. Therefore, we chose a Wilcoxon signed rank test to test for differences between simulated site results. Additionally, to give an estimate of the potential variability in metric and index scores due to misclassification, 95 % confidence intervals around the mean simulated score were derived from the 2.5th and 97.5th percentile of the metric and index scores distribution. The mean was chosen over the median as a conservative estimate of that distribution. Finally, the difference between original naive and simulated scores determined the bias of the naive assessment.

## Results

### Naive multi-metric index results

**Fig. 3:**
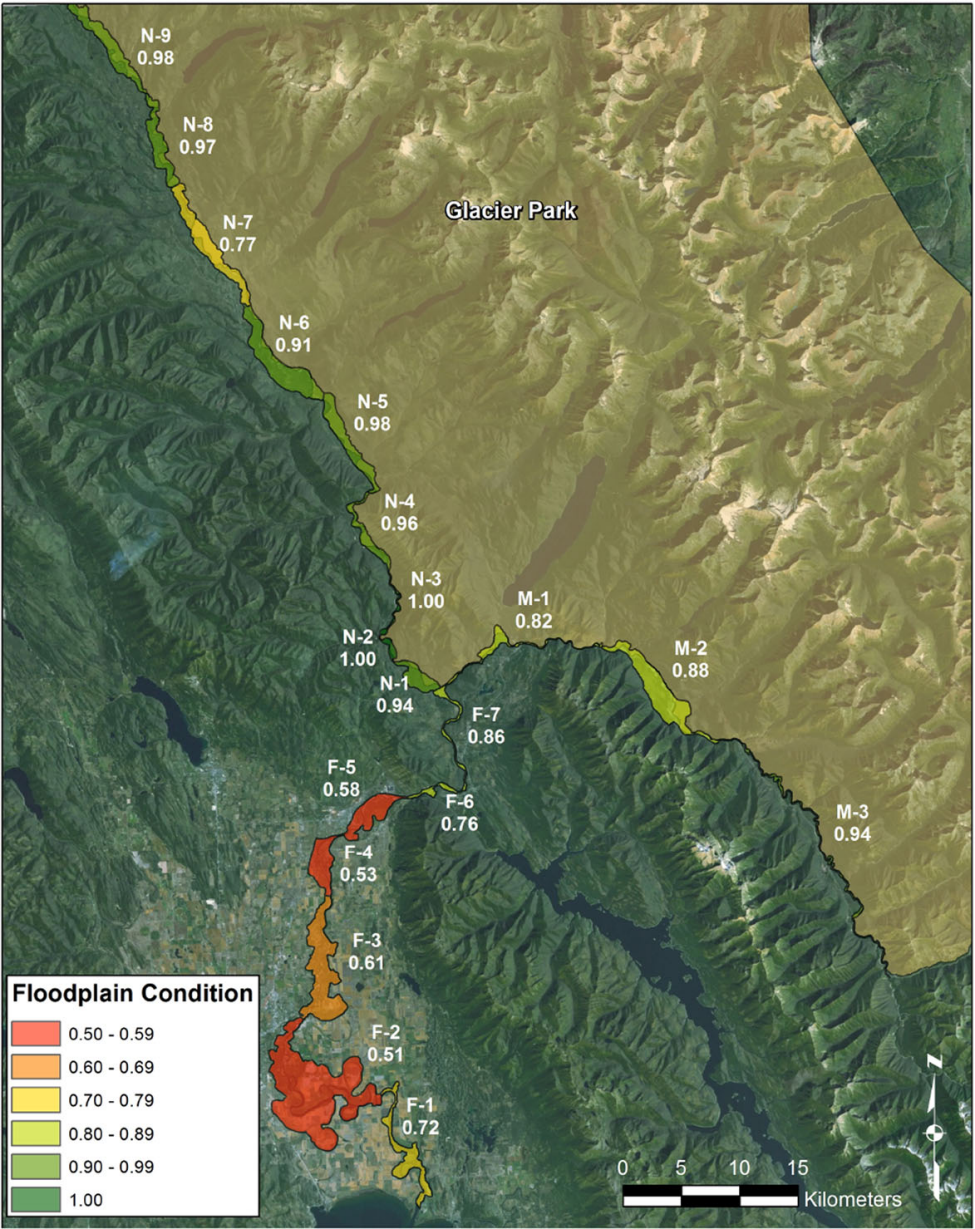
Synoptic map of Flathead River MMI scores

Typical of most MMIs, the initial results of this model were reported assuming that the input data was free from error (naive results). The final naive index scores articulated in the synoptic map (Fig. 3) closely matched the land-use/land-cover gradient across the study area (Fig. 2). Areas with intact, unmanaged lands scored in the upper index range (>0.90), areas with a mix of low-intensity agriculture and unmanaged lands scored in the middle range (~0.70–0.80), and areas with a mix of high- and low-intensity residential, agriculture, and unmanaged lands scored toward the lower end of the range (0.50–0.70).

### Map classification resolution

Aggregating land-cover groups lowered the resolution of thematic classifications in the original dataset from 16 classes to five classes for the perturbation map (Table 1) and two classes for the fragmentation map (Table 2). The 2006 NLCD map reported, at a national scale, an overall map accuracy of 78 % for maps classified into their standard 16 Level 2 land-cover classes (Wickham et al. 2013). For the perturbation metrics, the original 16 × 16 confusion matrix collapsed into a 5 × 5 matrix, thereby decreasing thematic resolution and increasing overall accuracy to 90 %. For the fragmentation metrics, a 2 × 2 confusion matrix summarized the binary cover classes with an overall accuracy of 92 %.

### Confusion frequency simulation results

**Fig. 4:**
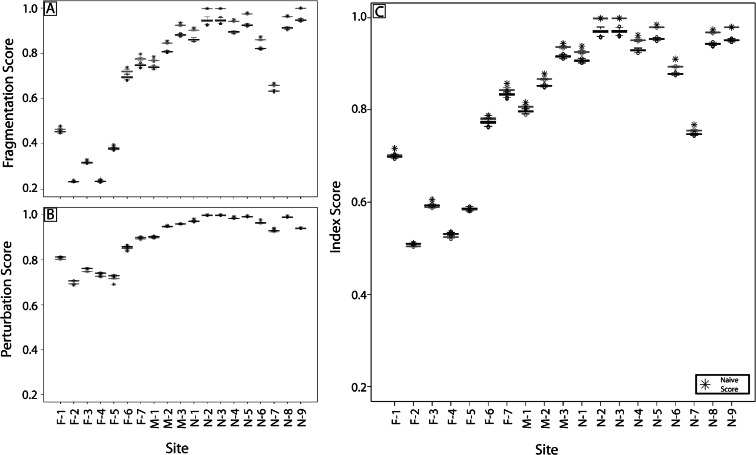
Naive data (*stars*) and distribution boxplots of simulated fragmentation (**a**), perturbation (**b**) scores averaged from the buffer and floodplain results, and index (**c**) scores with 10 % autocorrelation filters (*black*) and 20 % autocorrelation filters (*gray*)

For the error simulation model, user probability matrices (Tables 3 and 4) and autocorrelation filters were used in the confusion frequency simulations to provide a distribution of metrics and index scores, with 95 % confidence intervals (Fig. 4).^1^ The simulated and naive results closely match the LULC gradient across the study area (Fig. 2). A pairwise Wilcoxon signed rank test was applied to all simulated index sites using both the 10 and 20 % autocorrelation filter under the null hypothesis that there were no differences between the simulated sites. For sites N-2 and N-3, there was very strong evidence that they have the same mean index score (*p* value equal to 1.0) using the 10 % filter, but there was strong evidence that all sites were different (*p* value < 0.001) using the 20 % filter. Sites N-2 and N-3 both had naive score of 1.0 and all other naive scores were different. All remaining sites failed to support the null hypothesis, showing strong evidence of a difference between sites (*p* value < 0.001) for both filters.

### Sensitivity of simulated results to land cover

**Fig. 5:**
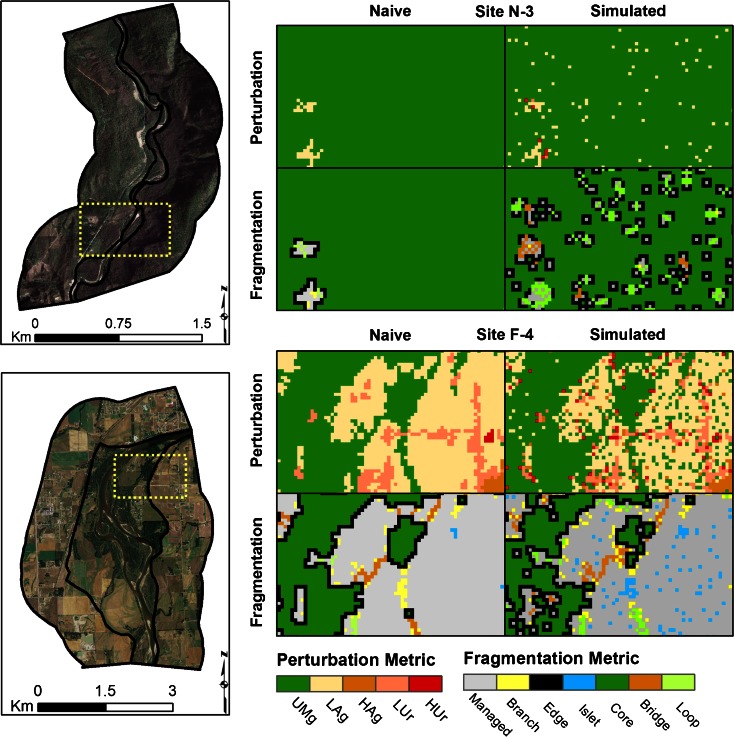
Perturbation and fragmentation maps for sites N-3 (*above*) and F-4 (*below*). Sample maps represent area demarked by *yellow box* in site maps. Naive maps are derived from original NLCD data and simulated maps are a realization from a single iteration of the CFS error model

Information from two sites with very different land covers (N-3 and F-4) provided an illustrative example of assessment metrics and index responses to map misclassifications. Site N-3 is located adjacent to Glacier National Park and is classified in the original NLCD map as 99.7 % unmanaged lands and 0.3 % low-intensity agriculture (Fig. 5 and Table 5), with a human population density of 3 people per kilometer. Site F-4 is located in the Kalispell Valley and contains a portion of the town of Columbia Falls, MT and nearby agricultural activities. The original land-use intensity classified this site as 22.6 % unmanaged lands, 42.2 and 19.8 % low- and high-intensity agriculture, respectively, and 13.6 and 1.8 % low- and high-intensity urban, respectively (Fig. 5 and Table 5), with a human population density of 639 people per kilometer. The landscape pattern structural classes in the two sites (Table 6) also reflect the land-use distributions. Site N-3 received metric and index scores of 1.0 for the naive assessment consistent, with its nearly contiguous cover of unmanaged lands (Table 7). Site F-4 scored 0.61 for the naive index score consistent, with its urban and agricultural land use mixed with patchy unmanaged land cover.

**Table 5:** Percent of land-cover classes from the original and simulated maps for sites N-3 and F-4

		Percent cover of perturbation classes				
		Unmanaged lands	Low-intensity agriculture	High-intensity agriculture	Low-intensity urban	High-intensity urban
Site F-4	Original	22.67	42.23	19.76	13.56	1.79
	Simulation	31.51	35.25	18.61	10.25	4.39
Site N-3	Original	99.73	0.27	–	–	–
	Simulation	97.58	2.37	–	0.01	0.04

**Table 6:** Percent of landscape pattern structural classes from the original and simulated maps for sites N-3 and F-4

		Percent cover of landscape pattern structures classes						
		Core	Edge	Loop	Bridge	Branch	Islet	Managed lands
Site F-4	Original	11.66	6.63	0.47	0.53	2.04	1.34	77.33
	Simulation	7.89	7.28	1.14	1.90	2.82	4.55	74.43
Site N-3	Original	99.52	0.42	0.06	–	–	–	–
	Simulation	74.85	17.43	4.10	0.24	0.05	–	3.34

**Table 7:** Metric and index results for naive and simulated distribution for sites N-3 and F-4, including resulting bias

		Perturbation		Fragmentation		Index
		Buffer	Floodplain	Buffer	Floodplain	
Site F-4	Original	0.62	0.84	0.06	0.42	0.53
	Simulation	0.650 (±0.004)	0.831 (±0.004)	0.071 (±0.003)	0.402 (±0.007)	0.531 (±0.003)
	Bias	−0.030	0.009	−0.011	0.018	−0.001
Site N-3	Original	1.00	1.00	1.00	1.00	1.00
	Simulation	0.995 (±0.001)	0.996 (±0.002)	0.943 (±0.006)	0.947 (±0.020)	0.971 (±0.007)
	Bias	0.005	0.004	0.057	0.053	0.029

For illustrative purposes, a single simulation was performed using the 10 % autocorrelation filter to create the map realizations in Fig. 5. The simulated realization reflects potential errors along patch edges and salt and pepper errors within patches (Fig. 5). These simulated errors decreased the overall cover of unmanaged lands in Site N-3 by about 2.4 % as these pixels were reassigned to low-intensity agriculture and urban land cover (Table 5). These reassigned pixels were peppered across the landscape (Fig. 5) and changed the composition of the landscape pattern structural classes (Table 6). These changes resulted in a slight decrease in the buffer and floodplain perturbation metrics of 0.005 and 0.004, respectively, a larger decrease in the buffer and floodplain fragmentation metric scores of 0.057 and 0.053, respectively, and an overall decrease in the index from 1.0 to 0.97 (Table 7).

In the site F-4 simulation, numerous former agriculture and low-intensity urban pixels were reassigned to unmanaged lands, increasing the percent cover of unmanaged lands from 23 to 32 %. There was also a slight increase in the high-intensity urban cover from 2 to 4 %. These changes were along patch edges and peppered within the patches (Fig. 5). Although there was an increase in the cover of unmanaged lands, there was a decrease in continuous patch cover in these lands. Because the buffer areas originally had higher urban and agriculture cover, the redistribution of pixel classes in the simulation resulted in increased mean perturbation and fragmentation metric scores in the buffer by 0.030 and 0.011, respectively. However, the floodplain originally had higher cover of unmanaged lands, and, as in site N-3, the redistribution of pixel classes in the simulated map decreased cover of unmanaged lands, which decreased both mean perturbation and fragmentation metric scores in the buffer by 0.009 and 0.018, respectively. After the index calculation, changes in the metric scores were essentially eliminated, with no change between the naive and mean-simulated index that both scored 0.53 after rounding (0.534 and 0.531, respectively; Table 7).

### Metric and index bias

**Fig. 6:**
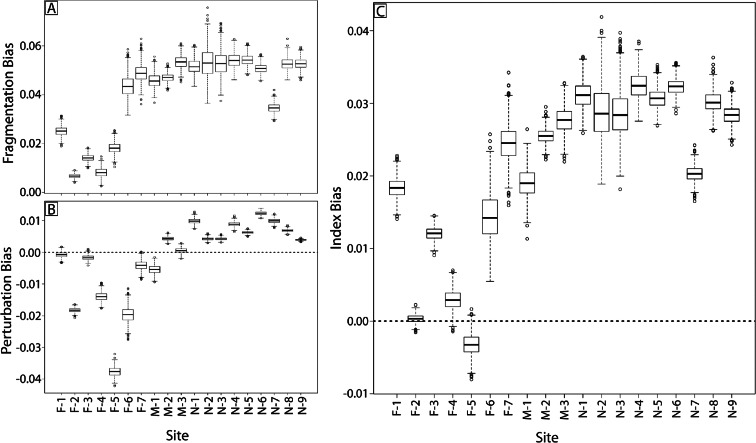
Distribution boxplots of bias of fragmentation (**a**), perturbation (**b**) scores averaged from the buffer and floodplain results, and index scores (**c**) for each assessment site

Bias between the naive index score and simulated results was determined using the 10 % autocorrelation filter. The difference between the naive score and total distribution of simulated scores indicated a bias in the estimation of the index and metrics resulting from misclassification (Fig. 6). The fragmentation metric showed a greater bias in sites dominated by unmanaged lands (Fig. 6a). Within the perturbation metric, sites with heterogeneous land use had a negative bias between the naive and simulated results (Fig. 6b). Collectively, there was a positive bias between most naive and simulated index results, with the highest bias in sites dominated by unmanaged lands (Fig. 6c). Our model showed that the fragment metric had a positive average bias of 3.91 %, with a maximum of 6.08 % for the floodplain area of N-6 and a minimum of −18.22 % for the buffer area of agriculture and urban site F-4. The perturbation metric had a negative average bias of −0.41 %, with a maximum of 2.16 % for the floodplain area of N-6 and a minimum of −11.02 % for the buffer area of urbanized site F-5. The naive index had positive average bias of about 2.39 %, with a maximum of 3.62 % at site N-6 and a minimum of −0.051 % at site F-5.

## Discussion

The confusion frequency simulation error model used here reveals that classification error affects assessment results in four important ways. First, naive results common to many large landscape assessment and monitoring efforts provide a biased estimate of habitat conditions compared to results that include errors. Second, depending on the land-cover composition of the assessment site, the magnitude and direction of this bias changes (Figs. 5 and 6 and Tables 6 and 7). Third, the magnitude and direction of the bias is independent for each metric (Fig. 6a, b). Finally, when these metrics are combined into an index, this bias is partially attenuated (Fig. 6c and Table 7).

All maps contain errors, and accuracy assessments provide insight into the extent and nature of misclassifications that are present. The confusion matrix is a foundation of classification accuracy assessment (Foody 2002). The NLCD 2006 map used here provides a confusion matrix associated with an accuracy assessment conducted at a continental scale only (Wickham et al. 2013). Fang et al. (2006) found that confusion matrices developed closer to the site of interest have much different error rates than regional or continental matrices. At any scale, the confusion matrix also has its own suite of inherent uncertainties. For instance, collection of reference data can also contain unmeasured sources of error (Foody 2002), and ground accuracy assessment teams may be inconsistent in the classification of mixed land cover in the assessment area or stratified random reference samples that may not capture spatially specific classification error (e.g., near patch edges). Additionally, although a confusion matrix is excellent at capturing thematic errors of omission and commission, it cannot capture all the non-thematic error that affects classification, such as misregistration of the image with ground data (Stehman 1997). Ultimately, obtaining a reliable confusion matrix and associated indices can be problematic (Pontius and Millones 2011). However, it currently remains the core accuracy assessment tool (Foody 2002). Regardless, the map user will be limited to the data provided unless they conduct their own accuracy assessment effort.

Confusion frequency simulation error models developed for categorical thematic maps use available information from the confusion matrix to account for errors resulting from misclassification (Fisher 1994; Hess and Bay 1997; Wickham et al. 1997; Langford et al. 2006). In the simulated realizations used here, pixels within the homogeneous unmanaged land cover are reclassified according to the user probability matrix, resulting in increased land-use heterogeneity and, thereby, lower assessment metric and index scores (Fig. 4). In contrast, sites with heterogeneous land uses are remixed to an alternative version of heterogeneity, resulting in a simulated map that may have higher or lower assessment scores depending on the ratio and spatial composition of managed to unmanaged lands in the original map (Figs. 2 and 4). Although this assessment did not have sites of homogeneous urban cover, such sites would be reclassified to have a higher cover of unmanaged lands according to the UPMs in Tables 3 and 4, which would raise the assessment metric and index scores.

Reducing error where possible is a first step to addressing uncertainty. The initial dataset provided an overall accuracy of 78 % for the 2006 NLCD continental-scale accuracy assessment. To create our assessment model, it was necessary to aggregate several of the land-cover categories into land-use groups, thereby lowering map classification resolution and resulting in increased overall accuracy to 90 % for the perturbation map and 92 % for the binary map. There were no radical departures between the between the naive and simulated results (Fig. 4), likely because of the input maps’ higher accuracies. However, if an alternative arrangement of the thematic input data were required to measure other aspects of the ecosystem, then the results would be different.

Because we intentionally did not collect site-specific map accuracy data, we remain ignorant of the spatial structure of the map error. However, we recognize that spatial autocorrelation affects the extent of misclassification within and between land-cover patches (Congalton 1988). When applied here, the 10 % spatial autocorrelation filter decreases the randomly located misclassifications within patches (salt and pepper error) and increases the misclassifications near patch boundaries. However, when applying the 20 % autocorrelation filter, this effect is exaggerated, resulting in simulated results that trend toward the naive results and an overall decrease in bias between the naive and simulated metric and index scores (Fig. 4). Without collecting the required local reference data to test the true relationships with autocorrelations, we felt it was best to be conservative in the face of uncertainty (Armstrong 2001) and applied the 10 % autocorrelation filter to the CFS error model. Ultimately, without an estimate of the structure of the spatial error, our simulation will likely contain its own misclassifications. However, our simulated values of ecological condition provide a more conservative estimate than our naive model results.

A remote-sensing product, such as the NLCD (MRLC 2013), is an appealing source of information for regional ecosystem assessment and monitoring. The NLCD provides thematic land-cover information and accuracy assessments that do not require the end-user to conduct the expensive and time-consuming (Foody 2002; Fang et al. 2006) necessary steps to process and analyze raw Landsat imagery or to collect additional accuracy assessment data (Homer et al. 2004, 2007). The above approach is not intended to be an assessment of the quality of the NLCD product; rather, it is intended to serve as a straightforward approach that could be used with any number of land-cover products.

Because we were interested in the uncertainty effects arising from a single source of input data and its impact on model outcomes, this case study did not address the other important sources of uncertainty that can manifest in the context and structure of ecosystem models (Walker et al. 2003; Refsgaard et al. 2007). Context refers to conditions and circumstances that frame the problem of interest from the perspective of the end-user (Walker et al. 2003). Our simplified case study assessed the overall condition of native floodplain habitats, which determined binning of the thematic data, weighting of the metrics sub-index scores, and the structure of the multi-metric model. If the model-building team and resource managers decide that other aspects of the system are important, such as the condition of floodplain wetlands, backwater channels, or forests, then structure of the binned input data would be different. Even with the model we chose, there were uncertainties built in to its structure and relationships between input data (including size of buffer, scale of assessment reaches, and binning of thematic data), metrics derived from these data, and their sub-index scores (Cressie et al. 2009). This epistemic uncertainty, due to imperfect knowledge, can be reduced through an increased understanding of how the modeled system works or refining of input data and its analysis. We recognize that any modeling effort should account for epistemic uncertainty due to model structure, and these efforts should be communicated to the end-user (Walker et al. 2003; Janssen et al. 2005; Refsgaard et al. 2007). However, for this effort, we were only interested in stochastic uncertainty due to inherent variability of the input data and its impact on the model outcome. Therefore, we accepted our simplified multi-metric case study for its consistent, albeit imperfect, structure.

### Implications of land-cover misclassification to resource decisions

Millions of dollars are spent annually in the U.S. on ecological monitoring, assessment, and restoration (Lovett et al. 2007; USEPA 2012). Landscape metrics and indices assist decision makers with allocating limited funds by prioritizing monitoring, protection, and restoration efforts (Hyman and Leibowitz 2000; Lausch and Herzog 2002; Steel et al. 2004; Hierl et al. 2008). Landscape metrics and indices are also frequently used to refine or test finer-scale monitoring and assessment tools (Stein et al. 2009; Rains et al. 2013). Also, quality thresholds are frequently used to trigger management actions and addressing the effects of classification error on assessment metric and index scores can assist decision makers in determining which sites are above or below such thresholds. However, the influence of classification accuracy on landscape indices has been largely ignored (Shao and Wu 2008). Without error assessment, applications of large landscape models for conservation decisions or finer-scale model development may be flawed.

Critical examinations of index-based approaches in the scientific literature (May 1985; Seegert 2000; Green and Chapman 2011) have addressed the shortcomings of metrics and indices in terms of sensitivity, calibration, and information loss. What are not seen in the literature are criticisms from the intended end-users of such models. Even if the scientific criticisms are accounted for, these models may fall into disuse when passed from scientist to end-user due to the overall lack of confidence in the assessment tool that results from uncertainty in its input data, the metrics it uses, and the output it creates. Tracking and reporting uncertainty is considered best practice in most remote-sensing and quantitative-modeling efforts. Although scientists have a general operational definition of uncertainty based on a model’s statistical properties, when applied to resource management, uncertainty in scientific outcomes potentially translates into a state-of-confidence that the decision maker has in its application. Policy makers view these uncertainties in association with their management goals and priorities (Walker et al. 2003).

The confusion frequency simulation error model used here is dependent on user probability matrices derived from the binned confusion matrix. If the original thematic map was binned differently to suit an assessment of different system attributes, then the UPMs, subsequent simulated realization of the maps, and resulting simulated metric and index would also change. By applying the CFS error model, we establish a distribution of potential metric and index scores and, therefore, bind the effect of the classification error. With our case study, the simulated results did not diverge greatly from the naive results (Fig. 4c). However, there are a few points of caution that should be kept in mind. First, the measured differences between the naive and simulated results of both the metrics and index imply that using naive results alone can be problematic. Second, although the CFS error model provides insight into potential land-cover realizations, changes in simulated assessment scores in area of homogeneous cover (e.g., Fig. 5 site N-3) can be also problematic. Finally, given the assessment scale and data resolution, both our naive and simulated results can distinguish between sites across the range of land use. However, to distinguish between sites with similar land use would require a different assessment tool to address local-scale disturbances. Nonetheless, providing information about error to the decision makers helps improve the state-of-confidence in the assessment tool.

Nevertheless, merely providing information on error within the model results does not necessarily assist the end-user in their ability to absorb that uncertainty into their decision. Interpretation tools, such as fuzzy sets and fuzzy operational rules, make it possible to formalize the knowledge of experts to provide information to assist the model end-user in areas where numerical data may be limited (Uricchio et al. 2004). Still, applying well-established approaches to characterize and interpret the degrees of uncertainty within data (e.g., rough sets, fuzzy sets, probability density functions) do not guarantee the assessment model will be used. As a tool, index-based assessments exist in the difficult area between science and policy (Turnhout et al. 2007), and scientists and model builders are not necessarily involved in the ultimate use of their product as a decision tool. Ideally, during the assessment tool development process, the science team works with the policy and stakeholders team to create a product that accounts for uncertainty and clearly articulates the limitations of the model in a manner that is easily understood by the end-user, so that the degrees and types of uncertainty in the model output can be reasonably absorbed into their decision process in a straightforward manner (Niemi and McDonald 2004; Turnhout et al. 2007).

Many of the historical advancements of assessment are well documented in the scientific literature. However, much of its development and application occurred in management settings (e.g., Adamus et al. 1987; Brinson et al. 1994; Hawkins et al. 2000; Hauer et al. 2002). Today, there are over 400 contemporary biological and structural assessment methods applied across a suite of environmental problems (Bartoldus 1999; Diaz et al. 2004; Fennessy et al. 2004; Böhringer and Jochem 2007). As the ease of access to classified Landsat products and geographic information tools increase, the number of landscape assessment metrics will likely expand as tools are developed to address a multitude of landscape-scale environmental problems. Each of these new metrics will have their unique sensitivity to classification error. For instance, several authors have already found that some landscape metrics are more sensitive to classification error than others (Hess and Bay 1997; Wickham et al. 1997; Shao et al. 2001; Langford et al. 2006). As our work has shown, metrics also respond differently to classification error across disturbance gradients associated with changes in LULC in each assessment site. Incorporating error sensitivity tests into the assessment model building process can help determine the level of classification errors that can be tolerated for existing and new landscape metrics and subsequent indices (Shao and Wu 2008).

## Conclusion

Our results elucidate the potential bias between the more common naive approach to ecological assessment and an approach that includes error. We show an increase in overall map accuracy as the 16 land-cover categories in the original NLCD thematic map was aggregated into the five land-use groups for the perturbation map and the two land-cover groups for our fragmentation map. These aggregated maps inform probabilities of misclassification within a confusion frequency simulation error model. The assessment metrics within our multi-metric index respond in different ways to map error depending on the land-cover pattern of each assessment site. When combined into an index, it appears that naive scores slightly over-estimate ecological quality within sites comprised of contagious unmanaged lands that are associated with higher quality floodplains. Additionally, the naive scores could potentially underestimate the quality in more disturbed sites comprised of heterogeneous land uses. Naive approaches are easier to implement. However, recognizing that using such an approach is biased may help with the end-user’s state-of-confidence in the landscape assessment tool.

## Appendix

### Confusion frequency simulation metric results

Using the confusion frequency simulation, each pixel retained its class assignment or was reassigned according to an outcome of a uniform random draw between 0 and 1 that was adjusted by the spatial autocorrelation filter. One thousand simulations of each metric were performed and an index score was calculated per iteration. These simulations provide a distribution of index scores and a 95 % confidence interval given the probabilities of class assignment (Tables 8, 9, and 10). The simulated data are provided in three significant digits to demonstrate the limitations of confidence intervals. The naive results are provided for comparison purposes and are reported in two significant digits, which is a general precision standard for most 0–1 MMI results.

**Table 8:** Perturbation metric results and confidence intervals from the 1000 Monte Carlo confusion frequency simulations and naive results for comparison

	Buffer perturbation			Naive score	Floodplain perturbation			Naive score
Site	2.50 %	50 %	97.50 %		2.50 %	50 %	97.50 %	
F-1	0.705	0.707	0.709	0.69	0.914	0.917	0.920	0.93
F-2	0.620	0.622	0.624	0.58	0.788	0.790	0.791	0.79
F-3	0.658	0.660	0.662	0.64	0.861	0.863	0.865	0.88
F-4	0.646	0.650	0.653	0.62	0.827	0.831	0.835	0.84
F-5	0.652	0.655	0.659	0.59	0.798	0.802	0.806	0.79
F-6	0.856	0.859	0.862	0.83	0.847	0.857	0.865	0.85
F-7	0.873	0.875	0.877	0.86	0.919	0.925	0.930	0.93
M-1	0.939	0.941	0.942	0.94	0.860	0.865	0.870	0.86
M-2	0.981	0.982	0.982	0.98	0.912	0.914	0.917	0.92
M-3	0.965	0.966	0.967	0.96	0.948	0.951	0.954	0.95
N-1	0.977	0.979	0.980	0.98	0.960	0.963	0.966	0.98
N-2	0.994	0.995	0.996	1.00	0.994	0.996	0.997	1.00
N-3	0.994	0.995	0.995	1.00	0.994	0.996	0.998	1.00
N-4	0.994	0.994	0.995	1.00	0.969	0.972	0.975	0.99
N-5	0.992	0.992	0.993	1.00	0.985	0.987	0.988	0.99
N-6	0.979	0.979	0.980	0.99	0.947	0.949	0.951	0.97
N-7	0.961	0.962	0.963	0.97	0.894	0.897	0.899	0.91
N-8	0.995	0.995	0.996	1.00	0.979	0.981	0.982	0.99
N-9	0.880	0.881	0.881	0.88	0.993	0.994	0.994	1.00

**Table 9:** Fragmentation metric results and confidence intervals from the 1000 Monte Carlo confusion frequency simulations and naive results for comparison

	Buffer fragmentation			Naive score	Floodplain fragmentation			Naive score
Site	2.50 %	50 %	97.50 %		2.50 %	50 %	97.50 %	
F-1	0.165	0.168	0.170	0.17	0.733	0.740	0.747	0.79
F-2	0.077	0.079	0.081	0.07	0.387	0.390	0.392	0.41
F-3	0.092	0.094	0.097	0.09	0.535	0.540	0.545	0.57
F-4	0.068	0.071	0.074	0.06	0.395	0.402	0.409	0.42
F-5	0.251	0.255	0.260	0.26	0.494	0.501	0.508	0.53
F-6	0.709	0.715	0.722	0.75	0.657	0.676	0.692	0.72
F-7	0.713	0.717	0.722	0.76	0.766	0.780	0.792	0.83
M-1	0.845	0.849	0.852	0.90	0.620	0.631	0.640	0.67
M-2	0.920	0.924	0.928	0.98	0.685	0.691	0.697	0.73
M-3	0.893	0.896	0.899	0.95	0.857	0.866	0.876	0.92
N-1	0.898	0.905	0.912	0.96	0.807	0.817	0.826	0.87
N-2	0.932	0.942	0.952	1.00	0.925	0.947	0.967	1.00
N-3	0.937	0.943	0.949	1.00	0.926	0.947	0.965	1.00
N-4	0.937	0.942	0.947	0.99	0.838	0.849	0.859	0.90
N-5	0.933	0.938	0.942	0.99	0.903	0.911	0.918	0.97
N-6	0.890	0.895	0.899	0.95	0.743	0.749	0.754	0.80
N-7	0.804	0.809	0.813	0.85	0.454	0.460	0.465	0.48
N-8	0.939	0.945	0.949	1.00	0.873	0.881	0.889	0.93
N-9	0.941	0.946	0.951	1.00	0.940	0.947	0.954	1.00

**Table 10:** Index results and confidence intervals from the 1000 Monte Carlo confusion frequency simulations and naive results for comparison

Site	Simulated index score			Naive index results
	2.50 %	50 %	97.50 %	
F-1	0.695	0.698	0.701	0.716
F-2	0.509	0.510	0.511	0.510
F-3	0.591	0.593	0.595	0.605
F-4	0.528	0.531	0.534	0.534
F-5	0.583	0.586	0.589	0.583
F-6	0.767	0.773	0.779	0.788
F-7	0.828	0.833	0.838	0.858
M-1	0.793	0.797	0.800	0.816
M-2	0.851	0.853	0.855	0.878
M-3	0.913	0.916	0.920	0.944
N-1	0.903	0.907	0.911	0.938
N-2	0.963	0.970	0.977	0.999
N-3	0.963	0.971	0.977	0.999
N-4	0.926	0.930	0.933	0.962
N-5	0.951	0.954	0.957	0.985
N-6	0.876	0.878	0.881	0.911
N-7	0.745	0.747	0.749	0.768
N-8	0.941	0.944	0.946	0.974
N-9	0.949	0.951	0.954	0.980

## Footnotes

1 Mean and 95 % confidence interval for the metric and index scores are available in the Appendix.
